# Trusted but Not Fully Verified: Generative AI Use and Exposure to Errors in Clinical Documentation Among Healthcare Professionals

**DOI:** 10.3390/healthcare14213660

**Published:** 2026-10-28

**Authors:** Carla Aurelia Stoiacovici, Alina Doina Tănase, Karina Cristina Marin, Adrian Cosmin Ilie

**Affiliations:** 1 Doctoral School, “Victor Babeș” University of Medicine and Pharmacy Timișoara, Eftimie Murgu Square 2, 300041 Timișoara, Romania; carla.stoiacovici@umft.ro; 2 Discipline of Professional Legislation in Dental Medicine, Faculty of Dental Medicine, “Victor Babeș” University of Medicine and Pharmacy Timișoara, Eftimie Murgu Square 2, 300041 Timișoara, Romania; tanase.alina@umft.ro; 3 Center for Medical Legislation, Malpractice, and Research Ethics, Faculty of Dental Medicine, “Victor Babeș” University of Medicine and Pharmacy Timișoara, Eftimie Murgu Square 2, 300041 Timișoara, Romania; 4 Department of Otorhinolaryngology, “Victor Babeș” University of Medicine and Pharmacy Timișoara, Eftimie Murgu Square 2, 300041 Timișoara, Romania; 5 Division of Public Health and Management, Department III Functional Sciences, “Victor Babeș” University of Medicine and Pharmacy Timișoara, Eftimie Murgu Square 2, 300041 Timișoara, Romania; ilie.adrian@umft.ro

**Keywords:** generative artificial intelligence, large language models, clinical documentation, patient safety, verification, trust, health personnel, cross-sectional studies

## Abstract

**Highlights:**

**What are the main findings?**

- Nearly half of surveyed professionals (44.4%) used generative AI for clinical documentation at least weekly, mostly for discharge summaries and referral letters; they verified a mean of 70% of outputs line by line, and 64% had encountered a clinically relevant error; higher trust was associated with less complete verification.
- Regular use was independently associated with trust in output accuracy, perceived usefulness, and lower perceived confidentiality risk, whereas AI literacy and formal training, which differed markedly between groups, were not independently associated once these attitudes were taken into account; after propensity-score weighting, differences in documentation burden and emotional exhaustion were small and imprecisely estimated.

**What are the implications of the main findings?**

- Verification of AI-drafted documents is an incomplete, trainable behavior that institutions should specify and monitor rather than assume.
- Governance should pair tool access with explicit guidance on permitted data inputs and confidentiality, since perceived risk, not error concern, is what separates users from non-users.

**Abstract:**

**Background/Objectives**: Generative artificial intelligence (GenAI) tools are entering clinical documentation ahead of institutional governance, and little is known about whether clinicians who rely on them also check their output, particularly in Central and Eastern Europe. This study characterized regular GenAI use for documentation, verification practices, and exposure to clinically relevant errors among healthcare professionals at a Romanian tertiary center, and identified the attitudes independently associated with regular use. **Methods**: In a cross-sectional survey of 126 physicians, residents, nurses, and public health specialists (46.0% of 315 invited), regular use was defined as at least weekly use for documentation during the preceding three months. Composites measured AI literacy, trust in output accuracy, perceived confidentiality risk, perceived usefulness, documentation burden, and intention; emotional exhaustion was measured with the Maslach Burnout Inventory. Correlates of regular use were examined with bootstrap-validated multivariable logistic regression, and differences in burden and exhaustion with propensity-score overlap weighting. **Results**: Regular use was reported by 56 participants (44.4%; 95% confidence interval [CI] 36.1–53.2), mostly with ChatGPT (89.3%) for referral and discharge letters (78.6%) and discharge summaries (69.6%). Users verified a mean of 70.3% (95% CI 65.6–75.0) of outputs line by line, and 36 (64.3%; 95% CI 51.2–75.5) had encountered at least one clinically relevant error. Among users, higher trust was associated with a lower proportion of outputs verified (Spearman ρ = −0.31; *p* = 0.020). Concern about factual errors was common in both groups (73.2% vs. 77.1%), whereas confidentiality concern, perceived insufficient training, and fear of liability were less frequent among users. Higher trust (adjusted odds ratio [OR] 2.36 per 10 points; 95% CI 1.34–4.15), higher perceived usefulness (OR 1.90; 1.03–3.50), and lower perceived risk (OR 0.49; 0.29–0.83) were independently associated with regular use (optimism-corrected area under the curve 0.905). After overlap weighting, differences in documentation burden (−4.8 points; 95% CI −12.1 to 2.6) and emotional exhaustion (−1.7; −5.9 to 2.5) were not statistically significant but were imprecisely estimated. **Conclusions**: Regular GenAI use coexisted with incomplete verification and frequent error exposure and was linked to trust and perceived usefulness; AI literacy and training were not independently associated with use once these attitudes were accounted for. Associations with documentation burden and emotional exhaustion were inconclusive. Institutions should treat verification as a distinct, trainable behavior and pair access with explicit confidentiality guidance.

## 1. Introduction

Large language models (LLMs) and the generative artificial intelligence (GenAI) applications built on them can be opened in a browser and used immediately, without institutional procurement, technical integration, or any formal decision to deploy them [1]. Because these tools accept free-text prompts and return fluent narrative output, they are particularly attractive for documentation, which occupies a large share of clinical time. Time-and-motion data show that ambulatory physicians spend nearly half of their working day on electronic health record and desk work, with documentation extending into personal time [2], and a scoping review found documentation burden to be measurable, pervasive across professions, and linked to reduced job satisfaction [3]. Clerical burden and the electronic environment have, in turn, been associated with physician burnout in large national samples [4]. It is therefore unsurprising that early deployments of ambient AI scribes were welcomed by clinicians who perceived less after-hours documentation and better patient interaction [5].

The output of these tools, however, is fluent whether or not it is correct. Early evaluations of GPT-4 in medicine emphasized its capacity to summarize and draft while cautioning that it produces confident but fabricated content, so that responsibility for verification rests entirely with the clinician [6]. In a blinded comparison of 100 hospital discharge summaries, LLM-generated narratives were rated equivalent to physician-written ones in overall quality but contained more errors, driven largely by omissions, and received higher potential-harm ratings [7]. Entering patient information into consumer tools also raises data-protection and consent questions that current regulation does not resolve [8,9], and regulatory scholars have argued that the pace of deployment has outstripped healthcare-specific oversight [10]. In the European Union, the Artificial Intelligence Act (Regulation (EU) 2024/1689) classifies AI systems used in healthcare according to risk and imposes transparency obligations on providers of general-purpose models, but it does not govern the informal, individual use of consumer chatbots by clinicians, which therefore remains a matter of data-protection law, professional standards, and institutional policy [11]. For a drafting tool, the safety-relevant behavior is therefore not adoption itself but what happens between generation of a draft and its incorporation into the patient record. Documentation was chosen as the focus of this study for three reasons: it is the most frequently reported real-world application of GenAI by clinicians [12,13]; it has the lowest barrier to entry, because drafting text requires no integration with clinical systems or institutional approval; and its output is directly auditable, because a draft can be compared with the source record. Diagnostic and therapeutic uses of GenAI raise distinct questions of decision support, accountability, and device regulation and were outside the scope of this study.

Human-factors research offers a vocabulary for this problem. Automation can be used, misused through over-reliance, or disused through unwarranted rejection, and each pattern has its own antecedents [14]. Appropriate reliance depends on trust that is calibrated to the actual capability of the system rather than on trust that is simply high or low [15]. Automation bias, the tendency to accept automated output and thereby commit errors of omission and commission, has been documented across clinical decision-support settings and is moderated by task complexity, user experience, and workload [16]. Conversely, people who witness an algorithm err may abandon it even when it outperforms them [17]. Trust has accordingly been identified as a central construct in clinicians’ relationships with AI, with both over-trust and under-trust carrying risks for safety and adoption [18]. Whether clinicians who regularly draft documents with GenAI verify that output, and whether their trust reflects experience of its errors, has rarely been measured directly.

Survey evidence on actual GenAI use by clinicians is accumulating but geographically concentrated. A national survey of general practitioners in the United Kingdom found that about one in five had used generative chatbots in practice, most often for documentation after appointments, in the absence of formal guidance [12]; a repeat survey of 1005 UK general practitioners in 2025 found that use had risen to one in four, with documentation still the most frequent application and 95% of users reporting no professional training [13]. In Germany, 45% of general practitioners surveyed in Saxony reported using some form of AI in practice, with lack of knowledge, data-security concerns, and legal uncertainty as the main barriers [19]. Outside Europe, a survey of healthcare workers in Saudi Arabia conducted shortly after the release of ChatGPT described a mixture of optimism about efficiency and reservation about accuracy and ethics [20], and a multinational convenience sample found that most professionals who had tried ChatGPT used it for writing rather than for direct clinical tasks [21]. These studies share three features that define the gap addressed here: they measured adoption and attitudes rather than what users do with the output they obtain; they rarely quantified verification or exposure to errors; and they were conducted predominantly among physicians in Western European or North American primary care or in online convenience samples, so that multi-professional hospital settings in Central and Eastern Europe, where language, legal culture, and digital infrastructure differ, remain unrepresented. An earlier cross-sectional study at the same tertiary center in Timișoara examined the adoption of AI tools in public health practice and identified technical knowledge and trust as positive, and legal concern as negative, correlates of adoption [22]. That study addressed AI broadly, as any AI-based tool used in public health work, sampled public health professionals, and did not examine documentation-specific behaviors, verification, error exposure, or clinician well-being. The present study was designed to fill these gaps by measuring, in a multi-professional hospital sample, not only whether GenAI is used for documentation but how much of its output is checked, how often errors are encountered, and whether use is associated with workload and exhaustion after adjustment for the characteristics that differ between users and non-users. The Unified Theory of Acceptance and Use of Technology (UTAUT) [23], which integrates eight earlier models of technology acceptance, positions performance expectancy (the belief that a technology improves job performance), effort expectancy (its perceived ease of use), social influence, and facilitating conditions as the proximal determinants of behavioral intention and use, complex-technology frameworks add the organizational conditions under which adoption succeeds or stalls [24], and AI literacy has been proposed as a modifiable determinant of appropriate use [25]. Which of these constructs distinguishes regular users of GenAI for documentation from their colleagues in an Eastern European hospital is not known.

The present study therefore examined regular GenAI use for clinical documentation among healthcare professionals at a Romanian tertiary academic center. The objectives were to estimate the prevalence and pattern of regular use; to describe verification practices and exposure to clinically relevant errors among regular users; to compare attitudes and perceived barriers between regular users and non-users; to identify the demographic, professional, and attitudinal characteristics independently associated with regular use; and to estimate whether regular use was associated with documentation burden and emotional exhaustion after accounting for measured differences between users and non-users.

## 2. Materials and Methods

### 2.1. Study Design and Setting

This was a single-center, analytical cross-sectional survey conducted at the “Pius Brînzeu” Clinical Emergency County Hospital Timișoara and its affiliated academic institution, the “Victor Babeș” University of Medicine and Pharmacy Timișoara, Romania. The hospital is the largest tertiary referral center in western Romania and employs attending physicians, residents in training, nurses, and public health specialists across medical, surgical, emergency, intensive care, and outpatient settings. Data were collected during a twelve-week enrolment window between January and March 2026. A cross-sectional design was chosen because the aims were to characterize the prevalence, pattern, and correlates of an existing behavior; temporal precedence between attitudes and use cannot be established with this design, and the adjustment methods described below were intended to reduce, not eliminate, confounding. The study is reported in accordance with the STROBE recommendations for cross-sectional studies.

The target population comprised healthcare professionals with at least six months of active clinical or public health practice at the center who produced written clinical or population-health documentation as part of their routine duties. The sample size of 126 participants was determined a priori for the comparison of composite attitudinal scores between regular users and non-users: assuming a standardized mean difference of 0.55, a two-sided α of 0.05, and an allocation ratio of approximately 45:55, 122 participants provide 85% power, and a target of at least 126 analyzable participants was set to provide a small margin above this figure. Of 145 questionnaires returned, 19 were excluded on the eligibility and completeness criteria described in Section 2.2 (Section 3.1 and Appendix A), leaving 126 participants; no record was excluded after entry into the analytic dataset. With 56 regular users and ten predictor terms, the sample provided 5.6 events per candidate predictor for the multivariable logistic model, which is below the conventional guidance of ten events per variable [26]; the implications are addressed by bootstrap optimism correction and by a penalized-likelihood sensitivity analysis (Section 2.4).

### 2.2. Participants and Definition of Regular Use

Eligible participants were attending physicians, senior residents (postgraduate year two or above), registered nurses, and public health specialists who were employed at the study center, had practiced for at least six months, and provided electronic informed consent. Exclusion criteria were purely administrative or managerial roles without documentation duties, extended leave during the enrolment window, prior participation in pilot testing of the questionnaire, and submission of a questionnaire with fewer than 80% of items completed. Recruitment used stratified invitation by professional category through the institutional e-mail system, with each invitation linked to a single-use token to prevent duplicate responses. Invitations were sent to all 315 professionals who met the eligibility criteria according to human-resources records (88 attending physicians, 74 residents, 112 nurses, and 41 public health specialists), and non-respondents received two reminders at two-week intervals. Participation was voluntary and uncompensated.

Regular GenAI use for clinical documentation was defined as self-reported deliberate use of at least one LLM-based tool (for example, ChatGPT, Gemini, or Microsoft Copilot) to draft, edit, summarize, or translate clinical documents at least once per week during the preceding three months. Because participants reported their own use of publicly available consumer tools, the specific model versions underlying these tools were not recorded; the versions in use were those available during the enrolment window (January–March 2026). Participants meeting this definition were classified as regular users; all others, including those with occasional or exploratory use, were classified as non-users. For descriptive purposes and for a sensitivity analysis, non-users were further distinguished into never-users and occasional users (any use for documentation during the preceding three months, but less than weekly). This operational definition was chosen to capture habitual integration into workflow rather than curiosity-driven experimentation. Prior formal AI training was defined as attendance at any structured course, workshop, or accredited module on AI in healthcare of at least four hours.

### 2.3. Measures and Data Collection

The questionnaire was developed from the UTAUT framework [23] (Section 1), in which performance expectancy, effort expectancy, social influence, and facilitating conditions are the proximal determinants of intention and use, and from the instrument used in the earlier adoption study at this center [22]. It was reviewed by three content experts and piloted with twelve professionals whose responses were excluded from the analysis. Demographic and professional covariates comprised age, sex, profession, years of experience, clinical unit, weekly working hours, awareness of institutional GenAI guidance, and self-estimated daily documentation time in minutes. Six attitudinal composites were derived from five-point Likert items, averaged within domain and linearly rescaled to 0–100, with higher scores indicating more of the named construct: AI literacy (eight items; Cronbach α = 0.88), trust in output accuracy (five items; α = 0.84), perceived confidentiality risk (five items; α = 0.81), perceived usefulness (five items; α = 0.86), documentation burden (six items; α = 0.83), and behavioral intention to use GenAI for documentation over the next twelve months (four items; α = 0.90). Example items were “I understand how large language models generate text” (AI literacy), “The clinical text produced by GenAI tools is factually accurate” (trust), “Entering patient information into GenAI tools puts patient confidentiality at risk” (perceived risk), “GenAI helps me produce documentation faster” (usefulness), and “The time I spend on documentation is excessive” (burden). The full questionnaire, with the wording of every item, its response format, and the composite to which it contributes, is provided in Appendix B (Table A2). The trust items referred to GenAI tools in general rather than to the respondent’s own use, so that they could be answered by all participants on the basis of any exposure, including occasional use, outputs produced by colleagues, or demonstrations; a “cannot judge” option was provided for every attitudinal item and was treated as missing. The three missing trust composites were all non-users who selected this option on most trust items. Among non-users, trust scores therefore reflect expectation of accuracy rather than experience with the tools, and this difference in referent is considered when interpreting the between-group comparison and the regression estimate (Section 4.5). Emotional exhaustion was measured with the nine-item emotional exhaustion subscale of the Maslach Burnout Inventory (MBI-EE) [27], scored 0–54. No formally validated Romanian version of the MBI-EE was available; a Romanian translation prepared for this study by forward and backward translation and reviewed by a bilingual occupational-health specialist was used, and its internal consistency in the present sample was α = 0.91. The MBI-EE items are licensed and are therefore not reproduced in Appendix B.

Regular users additionally reported their weekly hours of GenAI use, the tools used and the documentation tasks for which they used them (multiple-response items), their self-estimated documentation time saved per working day, the percentage of GenAI outputs that they verified line by line before incorporation into the record, and whether they had ever encountered a clinically relevant error in GenAI output. A clinically relevant error was defined for respondents in the questionnaire as “an error in the AI-generated text that, had it been entered into the record without correction, could have affected the diagnosis, treatment, follow-up, or safety of a patient (for example, a wrong drug or dose, an incorrect or fabricated finding, or the omission of a relevant diagnosis or result)”, as distinct from errors of style, formatting, or language. All participants completed eight binary attitude and barrier items, recorded as endorsed or not endorsed: concern about patient confidentiality, concern about factual errors or hallucinations, absence of institutional policy, insufficient training, limited Romanian-language performance, fear of medico-legal liability, poor integration with hospital software, and the belief that GenAI use should be disclosed to patients. Questionnaires were administered electronically on a secure institutional platform with embedded range and logic checks; two reverse-keyed items served as attention checks, and flagged records were reviewed before inclusion. All data were exported as a de-identified analytic dataset.

The study was approved as non-interventional, observational survey research by the Local Commission of Ethics of the “Pius Brînzeu” Clinical Emergency Hospital, Timișoara, Romania (Approval No. 316; 7 April 2025), and was conducted in accordance with the Declaration of Helsinki. Electronic informed consent was obtained before participation.

### 2.4. Statistical Analysis

Continuous variables are summarized as mean ± standard deviation (SD), or as median with interquartile range (IQR) where distributions were skewed, and categorical variables as counts with percentages; 95% confidence intervals (CI) for proportions were computed with the Wilson method and for means with the t distribution. Distributional assumptions were assessed with Shapiro–Wilk tests and Q–Q plots, and variance homogeneity with Levene’s test. Between-group comparisons of continuous variables used Welch’s *t*-test, with Hedges’ g and its 95% CI as the standardized effect size; categorical comparisons used Pearson’s χ^2^ test or Fisher’s exact test when any expected cell count was below five. Because the eight attitude and barrier items formed a family of related tests, their *p*-values were adjusted with the Benjamini–Hochberg false-discovery-rate procedure and are reported as q-values. Among regular users, the exploratory relationship between trust in output accuracy and the percentage of outputs verified line by line was examined with Spearman’s rank correlation (ρ) with a bootstrap 95% CI, and the verification percentage was compared between users who had and had not encountered a clinically relevant error with Welch’s *t*-test.

Independent correlates of regular use were identified with multivariable binary logistic regression including age (per 10 years), sex, profession, prior AI training, and the AI literacy, trust, perceived risk, and perceived usefulness composites (each per 10 points), reporting adjusted odds ratios (OR) with 95% CI. Multicollinearity was assessed with variance inflation factors (VIF), and a literacy × perceived-risk interaction was tested with a likelihood-ratio test. Model performance was quantified by the area under the receiver-operating-characteristic curve (AUC), Nagelkerke R^2^, the Hosmer–Lemeshow test, and the Brier score. Because the model was fitted and evaluated in the same sample, optimism-corrected AUC and calibration slope were obtained with 500 bootstrap resamples, and observed proportions of regular users were examined across quintiles of predicted probability. With 56 events and ten predictor terms (5.6 events per variable), the model falls below the conventional guidance of ten events per variable [26], although simulation work indicates that this threshold is not a strict boundary [28]. As a sensitivity analysis, the model was therefore refitted with Firth’s penalized likelihood, which reduces small-sample bias in the coefficients and their standard errors [29], and profile-likelihood 95% CIs are reported for that model.

To estimate the association of regular use with documentation burden and emotional exhaustion while addressing confounding by indication, a propensity score for regular use was estimated from age, sex, profession, prior training, daily documentation time, weekly working hours, AI literacy, and perceived confidentiality risk, and overlap weights (1 − propensity for users, propensity for non-users) were applied. Covariate balance was verified with standardized mean differences (SMD), and weighted mean differences were estimated with linear regression using heteroskedasticity-consistent (HC3) standard errors, both without and with additional covariate adjustment (doubly robust). E-values were computed to quantify the strength of unmeasured confounding required to explain away each doubly robust estimate. Because AI literacy and perceived confidentiality risk may be consequences as well as causes of use, the weighting analysis was repeated with a propensity score estimated from the six non-attitudinal covariates only (age, sex, profession, prior training, daily documentation time, and weekly working hours). A further sensitivity analysis excluded occasional users from the non-user group and repeated the logistic model and the weighted comparisons with never-users as the reference group.

Missing values were confined to three composites (trust, *n* = 3; documentation burden, *n* = 2; emotional exhaustion, *n* = 2; 0.5% of composite entries overall) and were assumed to be missing at random. Bivariate analyses used available cases. The multivariable model was fitted to the full analytic dataset (*n* = 126) with the seven missing composite entries imputed by single stochastic regression imputation using predictive mean matching, conditional on the other composites, the demographic and professional covariates, and regular-use status (the first of the chained-equations imputations described below, generated with a fixed random seed); as sensitivity analyses, the model was refitted across 20 datasets generated by multiple imputation by chained equations, with coefficients pooled by Rubin’s rules, and in complete cases. All tests were two-sided with statistical significance set at *p* < 0.05, and exact *p*-values are reported to three decimal places unless below 0.001. Analyses were performed in Python 3.11 with the NumPy 2.3, pandas 2.3, SciPy 1.17, statsmodels 0.15, and scikit-learn 1.8 libraries, and figures were produced with Matplotlib 3.10.

## 3. Results

### 3.1. Participants and Prevalence of Regular Use

Of the 315 eligible professionals invited, 145 (46.0%) returned a questionnaire: 39 of 88 attending physicians (44.3%), 41 of 74 residents (55.4%), 42 of 112 nurses (37.5%), and 23 of 41 public health specialists (56.1%). Nineteen respondents were excluded: seven did not meet the eligibility criteria on the screening items (fewer than six months of practice or no documentation duties), eight completed fewer than 80% of items, and four failed both attention checks and could not be resolved on review. The analytic sample therefore comprised 126 participants (40.0% of those invited), and its professional composition did not differ significantly from that of the invited population (χ^2^ = 3.94, 3 df; *p* = 0.268). Participant flow by stratum is given in Appendix A (Table A1). The 126 healthcare professionals analyzed comprised 34 attending physicians, 36 residents, 36 nurses, and 20 public health specialists. The mean age was 37.5 ± 9.8 years, and 89 participants (70.6%) were women. Seven composite entries were missing in six participants. Regular GenAI use for clinical documentation was reported by 56 participants (44.4%; 95% CI 36.1–53.2) and 70 (55.6%) were classified as non-users. Of the non-users, 23 (32.9% of non-users; 18.3% of the sample) reported occasional use for documentation during the preceding three months, and 47 had never used GenAI for documentation. Table 1 compares the demographic and professional characteristics of the two groups.

Regular users were younger than non-users (32.5 versus 41.5 years) and had less professional experience (7.1 versus 16.2 years; both *p* < 0.001). Residents accounted for 51.8% of users and 10.0% of non-users, so that 29 of 36 residents (80.6%) but only 12 of 34 attending physicians (35.3%), 9 of 36 nurses (25.0%), and 6 of 20 public health specialists (30.0%) were regular users (*p* < 0.001). Daily documentation time was longer among users (127.8 versus 115.4 min; *p* = 0.049). Regular use was reported by 19 of 33 participants with prior formal AI training (57.6%) and 37 of 93 without it (39.8%; *p* = 0.077). Sex, weekly working hours, clinical unit, and awareness of institutional guidance did not differ significantly between groups; only about one in five participants in either group was aware of any institutional guidance on GenAI.

### 3.2. Patterns of Use, Verification Practices, and Error Exposure

Table 2 and Figure 1 summarize the intensity of use, the tools and tasks involved, the perceived time savings, and the verification and error-exposure measures reported by the 56 regular users.

Regular users reported a median of 2.4 h of GenAI use per week (IQR 1.7–3.3; range 0.5–8.7). ChatGPT was used by 89.3%, Gemini by 30.4%, and Microsoft Copilot by 19.6%. The most frequent tasks were referral or discharge letters (78.6%), discharge summaries (69.6%), and translation of documents (62.5%); literature summaries and patient information material were each reported by 46.4%, and diagnosis or procedure coding by 14.3%. Users estimated a mean saving of 26.1 min of documentation time per day (95% CI 22.7–29.4).

On average, users reported verifying 70.3% (95% CI 65.6–75.0) of GenAI outputs line by line before entering them into the record, with substantial variation between individuals (SD 17.5). Thirty-six users (64.3%; 95% CI 51.2–75.5) had encountered at least one clinically relevant error in GenAI output. Incomplete verification and frequent error exposure thus coexisted within the same group of habitual users, whose predominant tasks were documents that enter the permanent record and are read by other clinicians. In an exploratory analysis among users, higher trust in output accuracy was associated with a lower proportion of outputs verified line by line (Spearman ρ = −0.31; bootstrap 95% CI −0.53 to −0.06; *p* = 0.020), whereas users who had encountered a clinically relevant error reported verifying a larger proportion of outputs than those who had not (74.1 ± 16.2% versus 63.5 ± 18.3%; mean difference 10.6, 95% CI 0.7 to 20.5; *p* = 0.037).

### 3.3. Attitudes and Perceived Barriers

Table 3 compares the attitudinal composites and emotional exhaustion between regular users and non-users, with standardized effect sizes.

Users had higher AI literacy, trust, perceived usefulness, and intention scores and lower perceived confidentiality risk than non-users (all *p* < 0.001), with large standardized differences (Hedges’ g 1.13 for literacy, 1.05 for trust, −0.90 for perceived risk, 0.82 for usefulness, and 1.76 for intention). Trust in output accuracy was nonetheless moderate in absolute terms even among users (mean 44.4 on the 0–100 scale). Documentation burden was lower among users in this unadjusted comparison (mean difference −7.8, 95% CI −13.3 to −2.2; *p* = 0.006), whereas the difference in emotional exhaustion was not statistically significant (−2.7, 95% CI −5.6 to 0.3; *p* = 0.073).

Table 4 and Figure 2 compare the endorsement of the eight attitude and barrier items between groups, with false-discovery-rate-adjusted q-values.

Concern about factual errors or hallucinations was the most frequently endorsed item and did not differ between users (73.2%) and non-users (77.1%; *q* = 0.698). By contrast, concern about patient confidentiality (30.4% versus 65.7%; *q* < 0.001), insufficient training (33.9% versus 62.9%; *q* = 0.005), and fear of medico-legal liability (25.0% versus 50.0%; *q* = 0.011) were endorsed about half as often by users as by non-users. The absence of an institutional policy, poor integration with hospital software, limited Romanian-language performance, and the view that GenAI use should be disclosed to patients were each endorsed by roughly half of both groups and did not differ significantly after adjustment for multiple testing.

### 3.4. Characteristics Independently Associated with Regular Use

The multivariable logistic regression model for regular use, its performance metrics, and the literacy × perceived-risk interaction test are reported in Table 5; the adjusted odds ratios are displayed in Figure 3.

After mutual adjustment, higher trust in output accuracy (adjusted OR 2.36 per 10 points; 95% CI 1.34–4.15), higher perceived usefulness (OR 1.90; 95% CI 1.03–3.50), and lower perceived confidentiality risk (OR 0.49 per 10 points; 95% CI 0.29–0.83) were independently associated with regular use. Older age (OR 0.34 per decade; 95% CI 0.13–0.90) and being a nurse rather than an attending physician (OR 0.14; 95% CI 0.02–0.87) were associated with lower odds. AI literacy (OR 1.25; 95% CI 0.71–2.19), prior formal training (OR 2.65; 95% CI 0.68–10.35), sex, the other professional categories, and the literacy × risk interaction (*p* = 0.238) were not significant once trust, perceived risk, and usefulness were in the model. Maximum VIF was 1.94, indicating no problematic collinearity.

The model discriminated well between users and non-users (apparent AUC 0.936; Brier score 0.099), and the Hosmer–Lemeshow test did not indicate lack of fit (*p* = 0.102). Bootstrap validation reduced the AUC to 0.905 (optimism 0.031) and yielded a calibration slope of 0.71 with calibration-in-the-large of 0.00, indicating some overfitting of the extreme predictions. Observed prevalence of regular use across quintiles of predicted probability was 3.8%, 0.0%, 48.0%, 80.0%, and 92.0%. At a predicted-probability cut-off of 0.48, sensitivity was 87.5% and specificity was 88.6%. In the Firth penalized model (Section 3.6), coefficients were shrunk modestly towards the null, but trust (OR 2.21 per 10 points; profile-likelihood 95% CI 1.31–3.86), perceived confidentiality risk (OR 0.52; 0.32–0.84), and perceived usefulness (OR 1.82; 1.02–3.31) remained independently associated with regular use, whereas AI literacy (OR 1.23; 0.73–2.10) and prior training (OR 2.38; 0.66–8.80) remained non-significant.

### 3.5. Documentation Burden and Emotional Exhaustion After Propensity-Score Weighting

Because regular users differed from non-users in age, profession, documentation load, literacy, and perceived risk, the unadjusted differences in documentation burden and emotional exhaustion in Table 3 were re-estimated after propensity-score overlap weighting (Table 6).

Overlap weighting achieved close balance on all eight covariates (largest absolute SMD < 0.01), at the cost of effective sample sizes of 29.1 users and 33.9 non-users. After weighting, the difference in documentation burden was attenuated and no longer statistically significant (−4.8 points, 95% CI −12.1 to 2.6; *p* = 0.204), as was the difference in emotional exhaustion (−1.7 points, 95% CI −5.9 to 2.5; *p* = 0.427). Doubly robust estimates were unchanged in direction and significance. The confidence intervals are compatible with both a modest benefit and no benefit of regular use, and the E-values (1.97 and 1.70) indicate that unmeasured confounding of only moderate strength could account for the point estimates.

### 3.6. Sensitivity Analyses

Table 7 summarizes the three sensitivity analyses. When the propensity score was estimated from the six non-attitudinal covariates only, balance remained acceptable (largest absolute SMD after weighting 0.08) and the effective sample sizes were larger (38.4 users and 44.7 non-users); the overlap-weighted differences in documentation burden (−6.1 points, 95% CI −13.0 to 0.8; *p* = 0.083) and emotional exhaustion (−2.1 points, 95% CI −6.1 to 1.9; *p* = 0.301) were slightly larger than in the primary analysis but remained non-significant, with intervals compatible with a modest benefit and with no benefit. When the 23 occasional users were excluded and regular users were compared with the 47 never-users, the adjusted associations of trust (OR 2.58 per 10 points; 95% CI 1.38–4.82), perceived confidentiality risk (OR 0.45; 0.25–0.80), and perceived usefulness (OR 2.04; 1.05–3.98) with regular use were somewhat stronger than in the primary model, AI literacy remained non-significant (OR 1.31; 0.71–2.42), and the weighted differences in burden (−5.3 points, 95% CI −13.1 to 2.5) and exhaustion (−1.9 points, 95% CI −6.4 to 2.6) were essentially unchanged. The Firth penalized model is described in Section 3.4. None of the sensitivity analyses altered the direction or interpretation of the primary findings.

## 4. Discussion

### 4.1. Principal Findings

In this tertiary academic center, nearly half of the surveyed healthcare professionals used GenAI for clinical documentation at least weekly, overwhelmingly through a general-purpose consumer chatbot and mainly for documents that enter the permanent record and are read by other clinicians. These habitual users reported checking roughly seven in ten outputs line by line, and almost two-thirds had already encountered a clinically relevant error in GenAI output. Among users, higher trust accompanied less complete verification, whereas prior error exposure accompanied more complete verification. Regular use was independently associated with higher trust in output accuracy, higher perceived usefulness, and lower perceived confidentiality risk, but not with AI literacy or formal training once these attitudes were taken into account, although both differed markedly between groups in unadjusted comparisons. Concern about factual errors was shared by users and non-users alike, whereas confidentiality concern, perceived training deficits, and liability fear were what most distinguished non-users. Finally, the lower documentation burden reported by users in unadjusted comparisons did not persist after balancing on measured characteristics, and the weighted estimates for both burden and emotional exhaustion were too imprecise to be conclusive.

The prevalence of regular use exceeded the roughly one in five general practitioners reporting any use in the United Kingdom survey [12], but differences in recruitment, professional composition, calendar period, and definitions of use preclude interpreting this contrast as a country effect. The findings extend the earlier work at this center [22], which surveyed public health professionals about the adoption of AI tools of any kind in public health practice and identified knowledge, trust, and legal concern as correlates of adoption. The present study differs from that work in its population (a multi-professional hospital sample dominated by physicians, residents, and nurses, recruited independently in a later enrolment window), in its exposure (GenAI use specifically for clinical documentation, with a frequency-based definition of regular use), in its outcomes (verification practice, error exposure, documentation burden, and emotional exhaustion, none of which was measured previously), and in its analytic approach (bootstrap-validated regression and propensity-score weighting). The one finding common to both studies, that trust and legal or confidentiality concerns distinguish users from non-users, therefore appears to hold across professional groups and across AI applications at this center. Their relevance is primarily to adoption and safety practices within comparable institutions rather than to population-wide estimates.

### 4.2. Verification and Error Exposure

The central observation of this study is that regular use, incomplete verification, and frequent error exposure coexisted within the same group of clinicians. A mean verification rate of 70% implies that, on average, nearly a third of GenAI-drafted content entered the drafting workflow without line-by-line review, even though most users had personally seen the tools produce clinically relevant errors. This pattern is consistent with the behavior that human-factors research describes as misuse of automation, in which reliance exceeds what the reliability of the system warrants [14], and with automation bias, in which users accept automated output and thereby fail to detect omissions or incorrect content [16]. It is also compatible with a more benign explanation: experienced users may concentrate verification on the tasks and passages they consider error-prone and skim the rest. The survey cannot distinguish calibrated selective review from complacency, and the verification measure does not establish that any unverified output was entered into the record unchanged. What it does establish is that verification is incomplete among the clinicians who use these tools most, which is precisely the group in which errors would propagate. The exploratory finding that higher trust was associated with less complete verification, whereas prior error exposure was associated with more complete verification, is consistent with the human-factors account in which reliance tracks trust and is recalibrated by experienced failures [15]; it links the two elements of the title directly, although the cross-sectional design cannot establish whether trust reduces checking or whether clinicians who check less come to trust more.

The frequency of reported error exposure is not surprising given the documentation tasks involved. In a blinded evaluation of hospital discharge summaries, LLM-generated narratives contained more errors than physician-written ones, mostly omissions, and were rated as having greater potential for harm, even though reviewers judged their overall quality equivalent [7]. Omissions are harder to detect than fabrications because a fluent, coherent summary gives no signal that something is missing, which argues for verification strategies that check the draft against the source record rather than reading the draft for plausibility. Discharge summaries and referral letters, the two most common tasks in the present sample, are also the documents most likely to be acted on by clinicians who did not witness the episode of care. The relevance of verification therefore scales with exactly the tasks for which GenAI is most used.

That most users continued to rely on GenAI after encountering errors runs counter to the algorithm aversion observed experimentally, in which seeing an algorithm err leads people to abandon it [17]. Several explanations are plausible. Users may regard occasional errors as an acceptable price for the time savings they perceive, they may believe that their own review catches the errors that matter, or the tasks for which they use GenAI may be ones in which errors are easily recognized. The moderate absolute level of trust among users, together with the near-universal concern about factual errors, suggests that these clinicians are not naively over-trusting; rather, their reliance appears to be a pragmatic judgment about usefulness under time pressure. Whether that judgment is well calibrated to the actual error rate of the tools in Romanian-language clinical documentation is unknown, and calibrated trust, not simply more or less trust, is what determines appropriate reliance [15,18].

### 4.3. Trust, Perceived Risk, and the Profile of the Regular User

In the adjusted model, the three attitudinal constructs that were independently associated with regular use were trust in output accuracy, perceived usefulness, and perceived confidentiality risk, whereas AI literacy and formal training were not. This was so despite a large unadjusted difference in literacy between groups (Hedges’ g = 1.13). This pattern is consistent with technology-acceptance theory, in which performance expectancy and trust are the proximal determinants of use and knowledge acts through them [23]. The null adjusted estimate for literacy should not be read as evidence that literacy is irrelevant: trust and perceived usefulness are plausible downstream consequences of literacy, so adjusting for them removes the part of the literacy association that operates through these attitudes, and the large unadjusted difference is compatible with literacy acting as a more distal determinant. A more cautious implication is that literacy or training interventions are likely to change behavior to the extent that they change how clinicians perceive the accuracy, usefulness, and data-handling risk of the tools. Because attitudes and use were measured simultaneously, the reverse pathway is equally plausible: regular use may raise trust and perceived usefulness through experience and lower perceived risk through habituation. Under either reading, the attitudes of regular users are the appropriate target for institutional messaging about verification and data protection, because those are the attitudes that accompany use.

The divergence between confidentiality concern and error concern deserves attention. Error concern was nearly universal and did not separate users from non-users, so it does not appear to deter use. Confidentiality concern, by contrast, was endorsed by two-thirds of non-users and fewer than a third of users, and perceived confidentiality risk remained one of the strongest independent correlates of non-use. Lower perceived risk among users is not necessarily more accurate; it may reflect misunderstanding of how consumer tools handle entered text. The questionnaire did not record whether users entered identifiable patient data into consumer tools, so the actual data-protection exposure of the behavior described here is unknown (Section 4.5). Ethical analyses of LLMs in medicine emphasize privacy and consent as unresolved problems [9], legal scholarship describes unsettled accountability and liability [8], and empirical work has shown that fluent output can reproduce harmful assumptions [30]. Institutions should make explicit which tools are approved, which data may be entered, who is responsible for verification, and how errors should be reported [31], rather than leaving individual clinicians to resolve these questions through their own risk tolerance. Only one in five participants in either group was aware of any institutional guidance, which indicates how far governance lagged behind practice.

The lower adjusted odds of regular use among nurses relative to attending physicians, after accounting for age and attitudes, may reflect several differences that the questionnaire did not capture. First, the documents for which GenAI was most used, discharge summaries and referral letters, are authored by physicians in the Romanian hospital system, whereas nursing documentation consists largely of structured flowsheets, care plans, and medication records that offer little scope for free-text drafting. Second, physicians and residents typically document at a workstation or on personal devices between patient encounters, whereas nurses document at the bedside or at shared ward terminals during shifts, where consumer chatbots are often blocked or impractical to open. Third, residents in particular encounter GenAI through academic work, examinations, and peer networks, and medical training in Romania involves more English-language material than nursing training, which matters because limited Romanian-language performance was a frequently endorsed barrier. Fourth, organizational expectations differ: physicians have discretion over how they produce narrative documents, whereas nursing documentation is more tightly standardized and audited. Finally, nurses in this sample were older on average and less often had prior AI training, and although age and training were adjusted for, residual differences in digital confidence are plausible. Experience with electronic-record implementation shows that technical, organizational, and individual barriers interact [32], and educational work on GenAI supports structured, role-specific competency development [33]. The strong age gradient and the high prevalence among residents indicate that regular use is already the norm among the clinicians who will constitute the future workforce, which strengthens the case for building verification standards into training rather than treating GenAI as an optional add-on.

### 4.4. Documentation Burden and Clinician Well-Being

Users estimated saving about 26 min of documentation time per day, yet after balancing on measured characteristics their documentation burden did not differ significantly from that of non-users, and the difference in emotional exhaustion was not statistically significant in any analysis. These results align with prospective evaluations of AI-drafted replies to patient messages, in which drafting assistance did not reduce the time physicians spent responding [34] and improved perceived burden without measurable time savings [35]. Ambient-scribe implementations have reported improvements in efficiency and perceived burden alongside variable editing experiences [5,36], but integrated ambient systems differ from the general-purpose tools and heterogeneous tasks described here. Task-level time savings may be real while total documentation burden is unchanged, because verification and editing absorb part of the time saved in drafting and because burden has organizational contributors that a drafting tool cannot address [37]. Romanian residents reported high levels of burnout during the pandemic years [38], and the present data give no indication that GenAI use has altered that picture. The weighted confidence intervals are compatible with modest benefit and with no benefit; they should not be read as evidence of equivalence. The sensitivity analysis that excluded attitudinal covariates from the propensity score yielded slightly larger point estimates with intervals that still included zero (Table 7), so the burden and exhaustion results are best described as inconclusive.

### 4.5. Limitations

The single-center cross-sectional design limits generalizability and prevents establishing temporal precedence between attitudes, use, verification, and error exposure; reverse causation and shared-method effects are plausible for all attitudinal associations. Participation was voluntary, the response rate was 46.0%, and professionals interested in AI may have been more likely to respond; although the professional composition of respondents matched that of the invited population, no other characteristics of non-respondents were available, so the direction and magnitude of selection bias cannot be quantified. Use, time saved, verification, and error exposure were self-reported and susceptible to recall and social-desirability bias. In particular, the verification measure was a self-estimated percentage rather than an observed behavior, and error exposure was recorded as ever having encountered a clinically relevant error, without quantification of error rates per document, error type, or consequences for patients. The questionnaire provided no objective measurement of document accuracy, actual time savings, or patient outcomes, and verification and error exposure were measured only among regular users, so their correlates could not be examined. The questionnaire did not ask whether identifiable patient data were entered into consumer tools, which is the behavior that determines the data-protection exposure discussed above, and it did not measure GenAI use for diagnostic or therapeutic decision-making, so the findings describe documentation use only. Trust was rated by all participants, but among non-users it necessarily reflects expectation rather than experience, and the trust–use association may partly capture this difference in referent rather than a difference in calibrated trust. Classifying the 23 occasional users with non-users may obscure differences between no exposure and intermittent use, although excluding them did not change the findings (Table 7), and the internal consistency of the locally developed composites and of the Romanian translation of the MBI-EE does not establish construct validity or measurement equivalence across professions.

The number of regular users was limited relative to the number of predictor terms (5.6 events per variable, below the conventional threshold of ten [26]), and the optimism-corrected calibration slope of 0.71 indicates overfitting despite good discrimination; the Firth penalized model led to the same conclusions, but wide intervals around the professional-category estimates reflect imprecision. The model is descriptive of this sample and is not intended for prediction in other settings. Overlap weighting improved measured balance but reduced the effective sample size and changed the target population to professionals with overlapping probabilities of use, and the inclusion of attitudinal covariates that may themselves be influenced by use complicates interpretation. E-values address unmeasured confounding only for the point estimates and do not establish robustness when intervals already include the null. Adjustment for multiple testing was applied to the barrier items but not to every comparison, and multiple imputation depends on the missing-at-random assumption.

### 4.6. Implications and Future Research

For institutions, the findings argue for treating verification as a distinct, trainable behavior rather than assuming that regular users check what they use. Practical measures include specifying that GenAI-drafted discharge summaries and referral letters be checked against the source record for omissions, defining permitted and prohibited data inputs, designating approved tools, and creating a low-threshold route for reporting GenAI-related errors so that local error patterns become visible. Training should target calibration of trust by exposing clinicians to realistic examples of omissions and fabrications in Romanian-language outputs, rather than general AI literacy alone, and should be embedded in residency given the prevalence of use among residents. Communication about confidentiality should be factual about how specific tools process entered text, because perceived risk currently separates users from non-users without any evidence that either group’s perception is accurate.

Prospective studies should measure attitudes, verification behavior, and workload before and after tool adoption, record the tool, task, and degree of electronic-record integration, and the nature of the data entered into the tools, and use independent document review to test whether self-reported verification corresponds to fewer omissions and errors in the final record. Measuring verification and error exposure in occasional users, and relating them to trust, workload, and task type, would clarify whether incomplete verification reflects calibrated selectivity or complacency. Controlled evaluations of verification training and governance interventions could then test whether changes in trust and perceived risk precede changes in behavior, and whether structured verification can be achieved without eroding the time savings that motivate use.

## 5. Conclusions

Regular GenAI use for clinical documentation was common among healthcare professionals at this Romanian tertiary center, concentrated among younger clinicians and residents, and directed mainly at discharge summaries and referral letters. Habitual users verified an average of seven in ten outputs line by line, and most had encountered clinically relevant errors, so incomplete verification and error exposure coexisted in the group best placed to propagate errors into the record. Trust in output accuracy, perceived usefulness, and lower perceived confidentiality risk distinguished regular users from non-users after adjustment, whereas AI literacy and training, although markedly higher among users, were not independently associated with use once these attitudes were taken into account. Among users, higher trust accompanied less complete verification. Whether regular use is associated with lower documentation burden or emotional exhaustion remained inconclusive after adjustment, because the weighted estimates were imprecise. Institutions should pair access to these tools with explicit verification standards, clear data-protection guidance, and prospective evaluation of documentation quality, treating safe use rather than uptake as the outcome that matters.

## Figures and Tables

**Figure 1:**
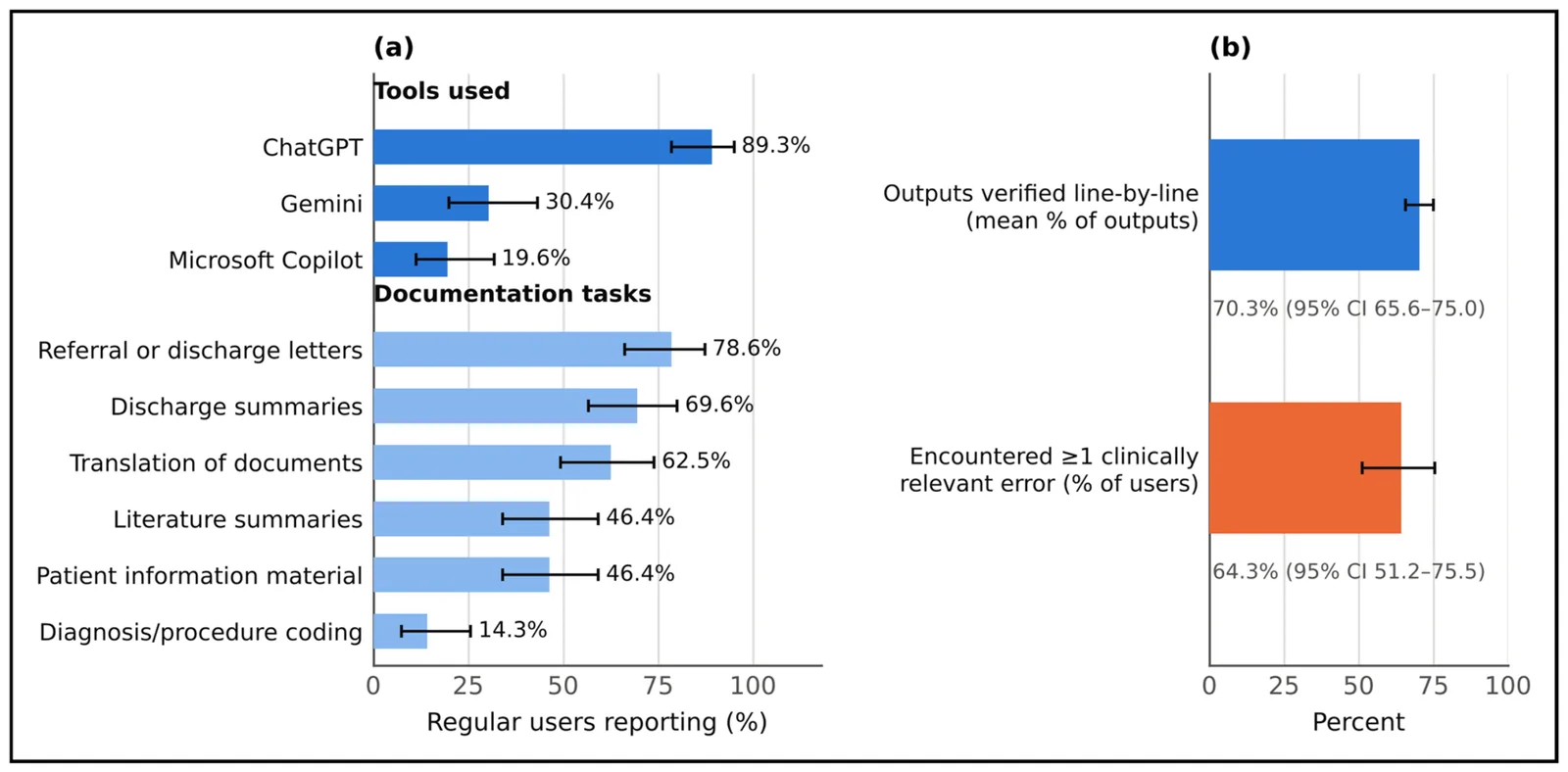
Patterns of GenAI use and safety-relevant practices among the 56 regular users. (**a**) Tools used and documentation tasks performed with GenAI (multiple-response items). (**b**) Mean percentage of GenAI outputs verified line by line before incorporation into the record and proportion of users who had ever encountered a clinically relevant error in GenAI output. Error bars are 95% confidence intervals (Wilson method for proportions; t-based for the mean). CI, confidence interval; GenAI, generative artificial intelligence.

**Figure 2:**
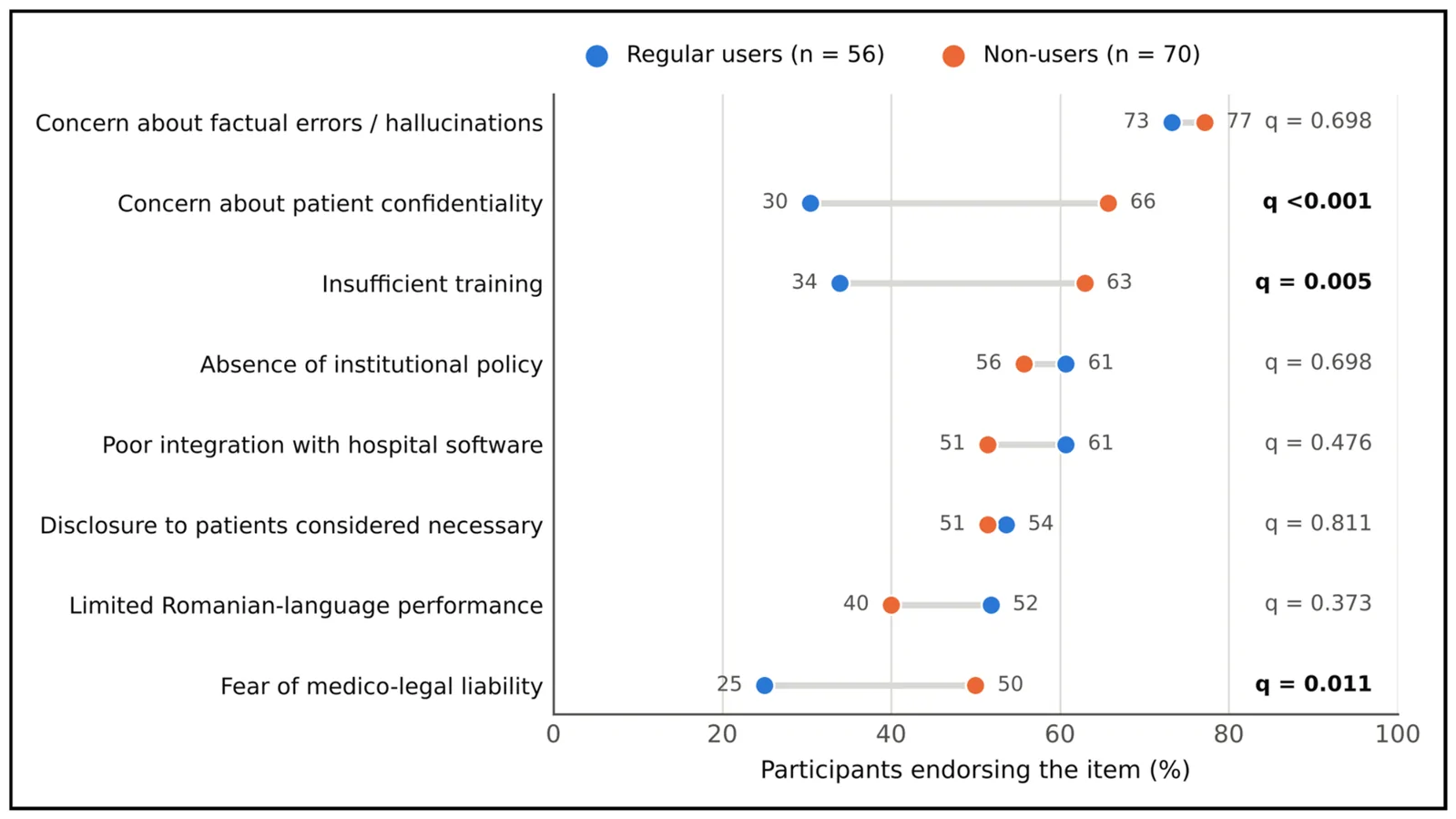
Endorsement of attitude and barrier items by regular users (*n* = 56) and non-users (*n* = 70) of GenAI for clinical documentation. Connected points show the percentage of each group endorsing the item; *q*-values are Benjamini–Hochberg false-discovery-rate-adjusted *p*-values from Pearson’s χ^2^ tests across the eight items, with *q* < 0.05 shown in bold. GenAI, generative artificial intelligence.

**Figure 3:**
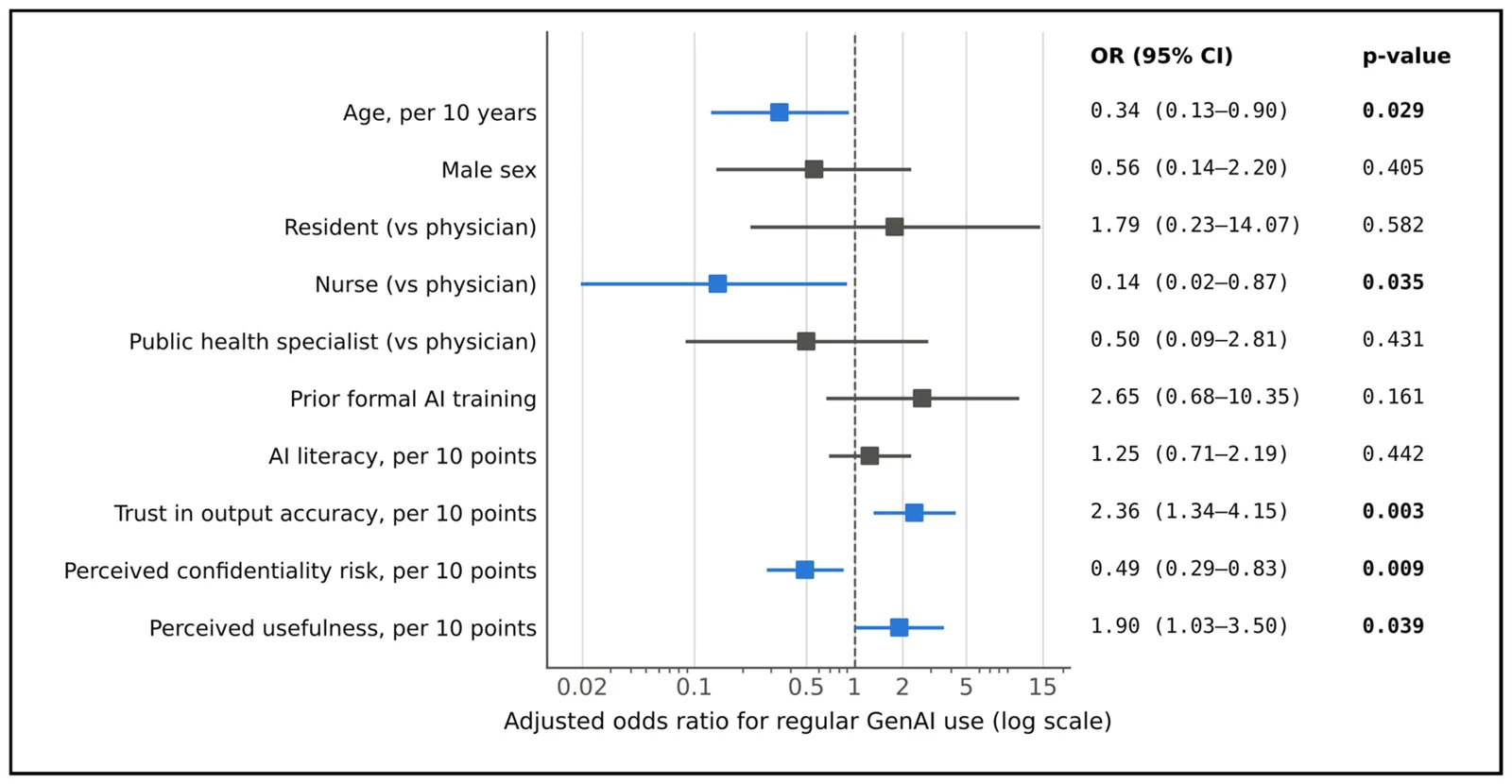
Forest plot of adjusted odds ratios (squares) with 95% confidence intervals (horizontal lines) from the multivariable logistic regression model for regular GenAI use (Table 5), plotted on a logarithmic scale. Predictors with *p* < 0.05 are shown in blue. AI, artificial intelligence; CI, confidence interval; GenAI, generative artificial intelligence; OR, odds ratio.

**Table 1:** Demographic and professional characteristics of participants by regular GenAI use for clinical documentation (*n* = 126). Continuous variables are mean ± SD and were compared with Welch’s *t*-test; categorical variables are *n* (%) and were compared with Pearson’s χ^2^ test.

Characteristic	Regular Users (*n* = 56)	Non-Users (*n* = 70)	Test Statistic	*p*-Value
Age, years	32.5 ± 8.1	41.5 ± 9.1	t = −5.91	<0.001
Professional experience, years	7.1 ± 8.1	16.2 ± 8.7	t = −6.06	<0.001
Weekly working hours	49.5 ± 7.8	47.9 ± 7.0	t = 1.24	0.216
Daily documentation time, min	127.8 ± 36.6	115.4 ± 32.6	t = 1.99	0.049
Female sex, *n* (%)	41 (73.2)	48 (68.6)	χ^2^ = 0.32	0.570
Prior formal AI training, *n* (%)	19 (33.9)	14 (20.0)	χ^2^ = 3.12	0.077
Aware of institutional GenAI guidance, *n* (%)	11 (19.6)	15 (21.4)	χ^2^ = 0.06	0.806
Profession, *n* (%)			χ^2^ = 27.37	<0.001
°Physician	12 (21.4)	22 (31.4)		
°Resident	29 (51.8)	7 (10.0)		
°Nurse	9 (16.1)	27 (38.6)		
°Public health specialist	6 (10.7)	14 (20.0)		
Clinical unit, *n* (%)			χ^2^ = 4.76	0.190
°Medical wards	11 (19.6)	22 (31.4)		
°Surgical wards	16 (28.6)	10 (14.3)		
°Emergency/ICU	10 (17.9)	13 (18.6)		
°Outpatient/public health	19 (33.9)	25 (35.7)		

AI, artificial intelligence; GenAI, generative artificial intelligence; ICU, intensive care unit; min, minutes; *n*, number of participants; SD, standard deviation; t, Welch’s t statistic; χ^2^, chi-square statistic. Daily documentation time refers to self-estimated minutes per working day spent producing written clinical or population-health documentation.

**Table 2:** Intensity of use, tools, documentation tasks, perceived benefit, verification practice, and exposure to clinically relevant errors among regular users of GenAI for clinical documentation (*n* = 56).

Measure	Estimate	95% CI or Range
*Intensity of use and tools*		
°Weekly GenAI use, h/week, median (IQR)	2.4 (1.7–3.3)	0.5–8.7
°ChatGPT, *n* (%)	50 (89.3)	78.5–95.0
°Gemini, *n* (%)	17 (30.4)	19.9–43.3
°Microsoft Copilot, *n* (%)	11 (19.6)	11.3–31.8
*Documentation tasks*		
°Referral or discharge letters, *n* (%)	44 (78.6)	66.2–87.3
°Discharge summaries, *n* (%)	39 (69.6)	56.7–80.1
°Translation of documents, *n* (%)	35 (62.5)	49.4–74.0
°Literature summaries, *n* (%)	26 (46.4)	34.0–59.3
°Patient information material, *n* (%)	26 (46.4)	34.0–59.3
°Diagnosis/procedure coding, *n* (%)	8 (14.3)	7.4–25.7
*Perceived benefit*		
°Self-estimated documentation time saved, min/day, mean ± SD	26.1 ± 12.5	22.7–29.4
*Verification and error exposure*		
°Outputs verified line by line before entry into the record, %, mean ± SD	70.3 ± 17.5	65.6–75.0
°Ever encountered a clinically relevant error in GenAI output, *n* (%)	36 (64.3)	51.2–75.5

CI, confidence interval (Wilson method for proportions; t-based for means); GenAI, generative artificial intelligence; h/week, hours per week; IQR, interquartile range; min/day, minutes per day; *n*, number of participants; SD, standard deviation. Tool and task categories were multiple-response items and do not sum to 100%. Time saved and the verification proportion were self-estimated by regular users.

**Table 3:** Attitudinal composite scores (0–100 unless stated) and emotional exhaustion in regular users and non-users, with mean differences, Hedges’ g, and Welch’s *t*-test.

Composite	Regular Users (*n* = 56)	Non-Users (*n* = 70)	Mean Difference (95% CI)	Hedges’ g (95% CI)	t (df)	*p*-Value
AI literacy	66.3 ± 12.3	51.8 ± 13.3	14.6 (10.1 to 19.1)	1.13 (0.75 to 1.51)	6.39 (121)	<0.001
Trust in output accuracy	44.4 ± 12.4	31.6 ± 11.8	12.8 (8.4 to 17.1)	1.05 (0.67 to 1.43)	5.80 (111)	<0.001
Perceived confidentiality risk	55.5 ± 12.5	67.5 ± 13.8	−12.0 (−16.6 to −7.3)	−0.90 (−1.27 to −0.53)	−5.11 (122)	<0.001
Perceived usefulness	56.8 ± 9.4	47.4 ± 12.6	9.4 (5.5 to 13.2)	0.82 (0.46 to 1.19)	4.77 (123)	<0.001
Documentation burden	51.5 ± 15.9	59.3 ± 14.9	−7.8 (−13.3 to −2.2)	−0.50 (−0.86 to −0.14)	−2.77 (110)	0.006
Emotional exhaustion (MBI-EE, 0–54)	21.7 ± 8.0	24.4 ± 8.6	−2.7 (−5.6 to 0.3)	−0.32 (−0.68 to 0.03)	−1.81 (119)	0.073
Intention to use	63.0 ± 13.7	37.4 ± 15.0	25.6 (20.6 to 30.7)	1.76 (1.35 to 2.18)	9.99 (122)	<0.001

AI, artificial intelligence; CI, confidence interval; df, Welch–Satterthwaite degrees of freedom; g, Hedges’ standardized mean difference; MBI-EE, Maslach Burnout Inventory emotional exhaustion subscale (0–54); *n*, number of participants; SD, standard deviation; t, Welch’s t statistic. Values are mean ± SD. Trust, documentation burden, and emotional exhaustion were available for 123, 124, and 124 participants, respectively.

**Table 4:** Endorsement of attitude and barrier items by regular users and non-users, with odds ratios, Pearson’s χ^2^ statistics, exact *p*-values, and Benjamini–Hochberg *q*-values.

Item Endorsed	Users, *n* (%)	Non-Users, *n* (%)	OR (95% CI)	χ^2^	*p*-Value	*q*-Value
Concern about patient confidentiality	17 (30.4)	46 (65.7)	0.23 (0.11–0.48)	15.56	<0.001	<0.001
Concern about factual errors/hallucinations	41 (73.2)	54 (77.1)	0.81 (0.36–1.83)	0.26	0.611	0.698
Absence of institutional policy	34 (60.7)	39 (55.7)	1.23 (0.60–2.51)	0.32	0.572	0.698
Insufficient training	19 (33.9)	44 (62.9)	0.30 (0.15–0.63)	10.41	0.001	0.005
Limited Romanian-language performance	29 (51.8)	28 (40.0)	1.61 (0.79–3.28)	1.74	0.187	0.373
Fear of medico-legal liability	14 (25.0)	35 (50.0)	0.33 (0.16–0.72)	8.18	0.004	0.011
Poor integration with hospital software	34 (60.7)	36 (51.4)	1.46 (0.72–2.98)	1.09	0.297	0.476
Disclosure to patients considered necessary	30 (53.6)	36 (51.4)	1.09 (0.54–2.20)	0.06	0.811	0.811

CI, confidence interval; *n*, number of participants; OR, odds ratio of endorsement among regular users relative to non-users (Woolf confidence intervals); *q*-value, Benjamini–Hochberg false-discovery-rate-adjusted *p*-value across the eight items; χ^2^, chi-square statistic. All χ^2^ tests have one degree of freedom.

**Table 5:** Multivariable binary logistic regression of characteristics independently associated with regular GenAI use for clinical documentation (*n* = 126; 56 events), with model performance and the literacy × perceived-risk interaction.

Predictor	β (SE)	Adjusted OR (95% CI)	z	*p*-Value
Age, per 10 years	−1.084 (0.498)	0.34 (0.13–0.90)	−2.18	0.029
Male sex	−0.582 (0.699)	0.56 (0.14–2.20)	−0.83	0.405
Resident (vs. physician)	0.580 (1.053)	1.79 (0.23–14.07)	0.55	0.582
Nurse (vs. physician)	−1.989 (0.945)	0.14 (0.02–0.87)	−2.11	0.035
Public health specialist (vs. physician)	−0.695 (0.882)	0.50 (0.09–2.81)	−0.79	0.431
Prior formal AI training	0.974 (0.695)	2.65 (0.68–10.35)	1.40	0.161
AI literacy, per 10 points	0.220 (0.287)	1.25 (0.71–2.19)	0.77	0.442
Trust in output accuracy, per 10 points	0.857 (0.289)	2.36 (1.34–4.15)	2.97	0.003
Perceived confidentiality risk, per 10 points	−0.713 (0.272)	0.49 (0.29–0.83)	−2.63	0.009
Perceived usefulness, per 10 points	0.642 (0.311)	1.90 (1.03–3.50)	2.06	0.039
*Model performance*				
°Likelihood-ratio χ^2^ (10 df)	91.18			<0.001
°Nagelkerke R^2^	0.690			
°Apparent AUC (95% CI)	0.936 (0.894–0.979)			
°Optimism-corrected AUC (500 bootstraps)	0.905			
°Optimism-corrected calibration slope	0.71			
°Hosmer–Lemeshow χ^2^ (8 df)	13.29			0.102
°Brier score	0.099			
°Maximum VIF	1.94			
°Literacy × risk interaction (LR test)	OR = 0.77			0.238

AI, artificial intelligence; AUC, area under the receiver-operating-characteristic curve; CI, confidence interval; df, degrees of freedom; GenAI, generative artificial intelligence; LR, likelihood ratio; *n*, number of participants; OR, odds ratio; R^2^, coefficient of determination; SE, standard error; VIF, variance inflation factor; vs., versus; z, Wald statistic; β, log-odds coefficient; χ^2^, chi-square statistic. The model was fitted to the full analytic dataset with missing composite entries imputed. In sensitivity analyses, Rubin-pooled estimates across 20 multiply imputed datasets were essentially identical (trust OR 2.32, 95% CI 1.32–4.08; perceived risk OR 0.49, 0.29–0.83; usefulness OR 1.89, 1.03–3.49; literacy OR 1.25, 0.72–2.20), as was the complete-case literacy estimate (OR 1.22; *n* = 123).

**Table 6:** Association of regular GenAI use with documentation burden and emotional exhaustion: crude, propensity-score overlap-weighted, and doubly robust estimates with E-values.

Outcome	Estimate	Difference (95% CI)	*p*-Value	E-Value
Documentation burden (0–100)	Crude	−7.6 (−13.0 to −2.2)	0.006	—
	Overlap-weighted	−4.8 (−12.1 to 2.6)	0.204	—
	Doubly robust	−4.8 (−13.2 to 3.6)	0.265	1.97
Emotional exhaustion (MBI-EE, 0–54)	Crude	−2.7 (−5.6 to 0.2)	0.066	—
	Overlap-weighted	−1.7 (−5.9 to 2.5)	0.427	—
	Doubly robust	−1.7 (−6.0 to 2.6)	0.441	1.70

AUC, area under the receiver-operating-characteristic curve; CI, confidence interval; GenAI, generative artificial intelligence; HC3, heteroskedasticity-consistent covariance estimator type 3; MBI-EE, Maslach Burnout Inventory emotional exhaustion subscale. Differences are users minus non-users, estimated by linear regression with HC3 standard errors (crude estimates therefore differ slightly from the Welch-based comparison in Table 3); E-values are reported for the doubly robust estimates only (—, not applicable). The propensity score was estimated from age, sex, profession, prior training, daily documentation time, weekly working hours, AI literacy, and perceived confidentiality risk (AUC 0.905); overlap weights ranged from 0.001 to 0.970, with effective sample sizes of 29.1 users and 33.9 non-users. The largest absolute standardized mean difference across the eight covariates was 1.14 before weighting and <0.01 after weighting. The doubly robust model additionally adjusted for all propensity-score covariates. The E-value is the minimum strength of association, on the risk-ratio scale, that an unmeasured confounder would need with both exposure and outcome to fully explain the doubly robust estimate.

**Table 7:** Sensitivity analyses for the multivariable logistic model of regular GenAI use and for the propensity-score-weighted comparisons of documentation burden and emotional exhaustion.

Analysis and Estimate	Primary Analysis	Sensitivity Analysis
*Firth penalized logistic regression (n = 126; 56 events): adjusted OR (95% CI)*		
°Trust in output accuracy, per 10 points	2.36 (1.34–4.15)	2.21 (1.31–3.86)
°Perceived confidentiality risk, per 10 points	0.49 (0.29–0.83)	0.52 (0.32–0.84)
°Perceived usefulness, per 10 points	1.90 (1.03–3.50)	1.82 (1.02–3.31)
°AI literacy, per 10 points	1.25 (0.71–2.19)	1.23 (0.73–2.10)
°Prior formal AI training	2.65 (0.68–10.35)	2.38 (0.66–8.80)
°Age, per 10 years	0.34 (0.13–0.90)	0.38 (0.15–0.92)
°Nurse (vs. physician)	0.14 (0.02–0.87)	0.17 (0.03–0.91)
*Propensity score without attitudinal covariates: overlap-weighted difference (95% CI)*		
°Documentation burden (0–100)	−4.8 (−12.1 to 2.6); *p* = 0.204	−6.1 (−13.0 to 0.8); *p* = 0.083
°Emotional exhaustion (MBI-EE, 0–54)	−1.7 (−5.9 to 2.5); *p* = 0.427	−2.1 (−6.1 to 1.9); *p* = 0.301
°Effective sample size, users/non-users	29.1/33.9	38.4/44.7
°Largest absolute SMD after weighting	<0.01	0.08
*Occasional users excluded (56 regular users* vs. *47 never-users; n = 103)*		
°Trust, adjusted OR per 10 points (95% CI)	2.36 (1.34–4.15)	2.58 (1.38–4.82)
°Perceived risk, adjusted OR per 10 points (95% CI)	0.49 (0.29–0.83)	0.45 (0.25–0.80)
°Usefulness, adjusted OR per 10 points (95% CI)	1.90 (1.03–3.50)	2.04 (1.05–3.98)
°AI literacy, adjusted OR per 10 points (95% CI)	1.25 (0.71–2.19)	1.31 (0.71–2.42)
°Documentation burden, weighted difference (95% CI)	−4.8 (−12.1 to 2.6)	−5.3 (−13.1 to 2.5)
°Emotional exhaustion, weighted difference (95% CI)	−1.7 (−5.9 to 2.5)	−1.9 (−6.4 to 2.6)

AI, artificial intelligence; CI, confidence interval; MBI-EE, Maslach Burnout Inventory emotional exhaustion subscale; *n*, number of participants; OR, odds ratio; SMD, standardized mean difference; vs., versus. The Firth model used the same ten predictor terms as the primary model (Table 5) with profile-likelihood CIs. The reduced propensity score was estimated from age, sex, profession, prior training, daily documentation time, and weekly working hours, and overlap weights were applied as in Table 6. In the analysis excluding occasional users, the logistic model was refitted with the same predictors and the propensity score was re-estimated from the eight covariates used in the primary analysis.

## Data Availability

The de-identified survey dataset and analysis code supporting the reported findings are available from the corresponding author upon reasonable request, subject to applicable institutional requirements for participant confidentiality.

## Abbreviations

The following abbreviations are used in this manuscript:

AI	Artificial intelligence
AUC	Area under the receiver-operating-characteristic curve
CI	Confidence interval
GCP	Good Clinical Practice
GenAI	Generative artificial intelligence
HC3	Heteroskedasticity-consistent covariance estimator type 3
ICH	International Conference on Harmonisation
ICU	Intensive care unit
IQR	Interquartile range
LLM	Large language model
LR	Likelihood ratio
MBI-EE	Maslach Burnout Inventory emotional exhaustion subscale
OR	Odds ratio
SD	Standard deviation
SE	Standard error
SMD	Standardized mean difference
STROBE	Strengthening the Reporting of Observational Studies in Epidemiology
UTAUT	Unified Theory of Acceptance and Use of Technology
VIF	Variance inflation factor

## Appendix A

Table A1 reports participant flow by professional category, from invitation to the analytic sample, in accordance with the STROBE recommendations.

**Table A1:** Participant flow by professional category.

Professional Category	Invited, *n*	Responded, *n* (%)	Excluded, *n*	Analyzed, *n* (% of Invited)
Attending physicians	88	39 (44.3)	5	34 (38.6)
Residents	74	41 (55.4)	5	36 (48.6)
Nurses	112	42 (37.5)	6	36 (32.1)
Public health specialists	41	23 (56.1)	3	20 (48.8)
Total	315	145 (46.0)	19	126 (40.0)

*n*, number of participants. Invitations were sent through the institutional e-mail system to all professionals meeting the eligibility criteria according to human-resources records, with two reminders. Exclusions comprised seven respondents who did not meet the eligibility criteria on the screening items (fewer than six months of practice or no documentation duties; two physicians, one resident, three nurses, one public health specialist), eight who completed fewer than 80% of items (two physicians, three residents, two nurses, one public health specialist), and four who failed both attention checks and could not be resolved on review (one physician, one resident, one nurse, one public health specialist). The professional composition of the analyzed sample did not differ significantly from that of the invited population (χ^2^ = 3.94, 3 df; *p* = 0.268).

## Appendix B

Table A2 reproduces the study questionnaire, with the wording of every item, its response format, and the composite to which it contributes.

**Table A2:** Study questionnaire: item wording, response formats, and scoring.

Section and Item	Wording (English Version of the Romanian Questionnaire)	Response Format/Scoring
*A. Demographic and professional characteristics*		
A1	Age	Years
A2	Sex	Female/male
A3	Professional category	Attending physician/resident (PGY ≥ 2)/registered nurse/public health specialist
A4	Years of professional experience since qualification	Years
A5	Clinical unit	Medical wards/surgical wards/emergency or ICU/outpatient or public health
A6	Average weekly working hours	Hours
A7	Approximately how many minutes per working day do you spend producing written clinical or population-health documentation?	Minutes
A8	Have you attended a structured course, workshop, or accredited module on AI in healthcare of at least four hours?	Yes/no (prior formal AI training)
A9	Are you aware of any guidance issued by your institution on the use of generative AI tools?	Yes/no
*B. Generative AI use for clinical documentation*		
B1	In the past three months, how often have you deliberately used a generative AI tool (for example ChatGPT, Gemini, or Microsoft Copilot) to draft, edit, summarize, or translate clinical documents?	Never/less than monthly/monthly/two or three times a month/weekly/several times a week/daily (regular use = weekly or more; occasional use = any use less than weekly)
B2 *	On average, how many hours per week do you use generative AI for documentation?	Hours (one decimal)
B3 *	Which tools do you use for documentation? (select all that apply)	ChatGPT/Gemini/Microsoft Copilot/other (specify)
B4 *	For which documentation tasks do you use generative AI? (select all that apply)	Referral or discharge letters/discharge summaries/translation of documents/literature summaries/patient information material/diagnosis or procedure coding/other
B5 *	Approximately how many minutes of documentation time does generative AI save you per working day?	Minutes
B6 *	Of the AI-generated text that you use, what percentage do you check line by line against the source information before entering it into the patient record?	0–100%
B7 *	Have you ever found a clinically relevant error in AI-generated text? A clinically relevant error is an error that, had it been entered into the record without correction, could have affected the diagnosis, treatment, follow-up, or safety of a patient (for example, a wrong drug or dose, an incorrect or fabricated finding, or the omission of a relevant diagnosis or result); errors of style, formatting, or language do not count.	Yes/no
*C. Attitudinal composites (each averaged within domain and rescaled to 0–100)*		
*AI literacy (8 items; α = 0.88)*		
L1	I understand how large language models generate text.	Likert 1–5 †
L2	I can explain why a generative AI tool may produce fabricated content (“hallucinations”).	Likert 1–5 †
L3	I know which types of tasks generative AI tools perform well and which they perform poorly.	Likert 1–5 †
L4	I can write prompts that produce the output I need.	Likert 1–5 †
L5	I know how the generative AI tools I might use store and process the text entered into them.	Likert 1–5 †
L6	I can recognize when generative AI output is likely to be unreliable.	Likert 1–5 †
L7	I keep up with developments in AI that are relevant to my work.	Likert 1–5 †
L8	I feel competent to use generative AI tools in my professional work.	Likert 1–5 †
*Trust in output accuracy (5 items; α = 0.84)*		
T1	The clinical text produced by generative AI tools is factually accurate.	Likert 1–5 †
T2	Generative AI tools reliably reproduce the information they are given without distorting it.	Likert 1–5 †
T3	I would trust a discharge summary drafted by generative AI after a brief read-through.	Likert 1–5 †
T4 (R)	Generative AI output needs to be checked word by word before any of it can be used.	Likert 1–5 †; reverse-scored; attention check
T5	Overall, I trust the accuracy of generative AI output for clinical documentation.	Likert 1–5 †
*Perceived confidentiality risk (5 items; α = 0.81)*		
R1	Entering patient information into generative AI tools puts patient confidentiality at risk.	Likert 1–5 †
R2	Text entered into consumer generative AI tools may be stored or used by the provider of the tool.	Likert 1–5 †
R3	Using generative AI for documentation could breach data-protection law (General Data Protection Regulation).	Likert 1–5 †
R4	Patients would object if they knew that their information had been entered into a generative AI tool.	Likert 1–5 †
R5	The confidentiality risks of generative AI outweigh its benefits for documentation.	Likert 1–5 †
*Perceived usefulness (5 items; α = 0.86)*		
U1	Generative AI helps me produce documentation faster.	Likert 1–5 †
U2	Generative AI improves the clarity and completeness of my documents.	Likert 1–5 †
U3	Generative AI reduces the effort of routine documentation tasks.	Likert 1–5 †
U4	Generative AI output is a useful starting point even when it needs editing.	Likert 1–5 †
U5 (R)	Using generative AI for documentation would make little difference to my work.	Likert 1–5 †; reverse-scored; attention check
*Documentation burden (6 items; α = 0.83)*		
D1	The time I spend on documentation is excessive.	Likert 1–5 †
D2	Documentation frequently extends into my personal time.	Likert 1–5 †
D3	Documentation reduces the time I can spend with patients.	Likert 1–5 †
D4	I find the documentation requirements of my work frustrating.	Likert 1–5 †
D5	The documentation systems I use are inefficient.	Likert 1–5 †
D6	Documentation is a major source of stress in my work.	Likert 1–5 †
*Behavioral intention (4 items; α = 0.90)*		
I1	I intend to use generative AI for documentation during the next twelve months.	Likert 1–5 †
I2	I expect my use of generative AI for documentation to increase.	Likert 1–5 †
I3	I would use generative AI for documentation if institutional guidance permitted it.	Likert 1–5 †
I4	I would recommend generative AI for documentation to colleagues.	Likert 1–5 †
*D. Attitude and barrier items*		
E1–E8	Which of the following apply to your view of generative AI for clinical documentation? (select all that apply): concern about patient confidentiality; concern about factual errors or hallucinations; absence of an institutional policy; insufficient training; limited performance in Romanian; fear of medico-legal liability; poor integration with hospital software; use of generative AI should be disclosed to patients.	Endorsed/not endorsed (eight binary items)
*E. Emotional exhaustion*		
MBI-EE 1–9	Nine items of the emotional exhaustion subscale of the Maslach Burnout Inventory [27], Romanian translation; items are licensed and not reproduced.	0 (never) to 6 (every day); summed 0–54

AI, artificial intelligence; ICU, intensive care unit; MBI-EE, Maslach Burnout Inventory emotional exhaustion subscale; PGY, postgraduate year; (R), reverse-keyed item; α, Cronbach’s alpha in the present sample. † Five-point Likert scale (1 = strongly disagree to 5 = strongly agree) with an additional “cannot judge” option. Items marked * were presented only to participants who reported any generative AI use for documentation in item B1 (B2–B7 were analyzed for regular users). Composite scores were computed when at least 80% of the items in a domain were answered; “cannot judge” responses were treated as missing. The two reverse-keyed items (T4 and U5) also served as attention checks: records in which both were answered in the same direction as the extreme of the positively keyed items were flagged and reviewed. The questionnaire was administered in Romanian; the English wording above is the authors’ translation.
